# Teenager Presenting With Chest Pain and ST-Segment Changes on Electrocardiogram After SARS-CoV-2 Illness: Early Repolarization vs. Acute Pericarditis

**DOI:** 10.7759/cureus.24654

**Published:** 2022-05-01

**Authors:** Richard Amoateng, Ibrahim Ahmed, Abraham Attah, Brent Hardman

**Affiliations:** 1 Internal Medicine, Allegheny Health Network, Pittsburgh, USA; 2 Internal Medicine, Mercy Catholic Medical Center, Philadelphia, USA; 3 Emergency Medicine and Internal Medicine, Allegheny Health Network, Pittsburgh, USA

**Keywords:** st-segment changes, chest pain, sars-cov-2, acute pericarditis, early depolarization

## Abstract

The ST-segment elevation is commonly associated with acute myocardial Infarction. However, there are other non-ischemic causes of ST-elevation. Severe acute respiratory syndrome coronavirus 2 (SARS-CoV-2) is a highly contagious illness that continues to plague the world since the first case was reported in China over two years ago. As cases of the diseases become rampant, we have learned more of its complications which can include cardiac and pericardial disease. We present a case report of a young African American male who presented with chest pain six weeks after being diagnosed with SARS-Cov-2 pneumonia. Electrocardiogram (EKG) showed ST-segment changes that were initially presumed to be acute pericarditis. The patient was initially treated with colchicine. After further workup and a second opinion, ST-segment changes were thought to be likely benign early repolarization changes rather than pericarditis. Differential diagnosis of ST-segment changes on EKG in the patient with chest pain is broad. Subtle findings on EKG are important in distinguishing these differentials and should be well known and understood.

## Introduction

ST-segment elevations are commonly associated with acute myocardial infarction. However, there can be non-ischemic causes of this electrocardiogram (EKG) abnormality, including pericarditis, benign early repolarization, takotsubo cardiomyopathy, printzmetal angina, left ventricle aneurysm, and some rarer causes [1]. It is important to be able to differentiate the cause of the ST-segment elevation quickly and often based just on EKG findings, especially when in an underserved area where resources may be scarce. With new diseases such as severe acute respiratory syndrome coronavirus-2 (SARS-CoV-2). It is important to keep a broad differential so as to not miss a new constellation of symptoms related to a new disease.

Severe acute respiratory syndrome coronavirus-2 (SARS-CoV-2) also known as COVID-19 is a novel pathogen belonging to the family of coronaviruses currently responsible for a global pandemic. The first cases were reported in Wuhan, China at the end of 2019 and has since rapidly spread to almost every corner of the planet. As the number of global cases continues to climb our understanding of this virus has also evolved. The virus presents with a wide range of symptoms including cough, fever, myalgia, headaches, dyspnea, sore throat, diarrhea, and loss of smell and taste among others [2]. The clinical course of the virus can range from asymptomatic, mild symptoms to critical illness [3]. Several complications have been associated with COVID-19 illness. Acute respiratory distress syndrome (ARDS) is the major respiratory complication seen in patients. Pulmonary emboli and acute strokes are thromboembolic phenomena also seen in COVID-19. Other complications include encephalopathy, cardiovascular complications such as arrhythmias, acute cardiac injury, cardiomyopathy, pericarditis, and cardiogenic shock [4-7].

Acute pericarditis is an inflammatory disorder of the pericardium that is a common cause of non-ischemic chest pain among hospitalized patients or those presenting to the emergency room [8,9]. Although the etiology of acute pericarditis in most clinical settings is unknown, acute pericarditis is frequently associated with viral illness [9]. Acute pericarditis has been reported to be associated with some COVID-19 patients in some case reports [10-15].

Early repolarization (ER) on the other hand has historically been associated with young healthy athletic individuals. It often is an incidental finding without clinical significance. Nonetheless, a small proportion of ER is associated with idiopathic ventricular fibrillation which does have clinical significance [6-17]. 

We present a case report of ST-segment elevation in a patient after subacute COVID-19 illness.

## Case presentation

A 19-year-old African American male presented to an urgent care clinic in an underserved community with the chief complaint of chest pain. He was diagnosed with COVID-19 pneumonia two months ago and was sick for about two weeks with cough, chest pain, and dyspnea, which resolved six weeks before presentation. The patient was unvaccinated against SARS-CoV-2 prior to his illness. He had been back to his baseline without any residual COVID-19 symptoms. He then presented to our clinic with new complaints of a two-day history of chest pain. He described it as a sharp pain in the middle of the chest that is exacerbated with a supine position or movements of the arms. His chest pain was relieved when he leaned forward. The pain began suddenly two days ago when he woke up from sleep. He described the pain as sharp without radiation but worsening with inspiration. He denied any dyspnea, fevers, cough, chest congestion, dizziness, lightheadedness, or leg swelling. He had no problem with exertion, or exercise intolerance, and of note was just starting a new exercise routine three days ago, which did include bench press and pectoral type activities. He denied any recent trauma, falls, or injury to the area.

The patient had no current medical issues, no medications, surgical history was significant for remote appendectomy. He reported occasional marijuana smoking, but no other illicit drug usage and was up to date with his vaccinations.

Examination showed stable vital signs: blood pressure of 138/75, a pulse of 77, afebrile at 99.0^o^F, respiratory rate of 16, oxygen saturation of 99% on the radial artery (RA). He was in no apparent distress and had no jugular vein distension (JVD), lower extremity edema, or rhonchi, rales, or crackles on the lung exam. Cardiac examination showed a normal S1 and S2 without a murmur, gallop, or rub, and normal peripheral pulses. The chest wall did have some tenderness over the lower part of both pectoral muscles that also was reproducible with the patient extending both arms in a chest press type motion.

Investigations

**Figure 1:**
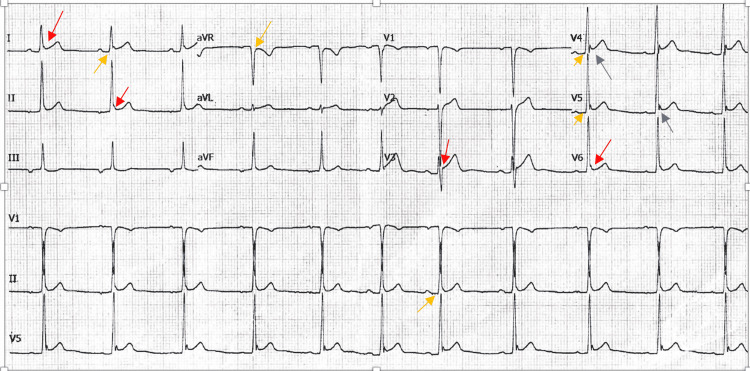
EKG on day 1 of presentation >1 mm J-point elevation/concave ST-segment elevation in leads I, II, V2, V3, V4, V5, V6. Orange arrows: PR depressions in leads I, II, V4, V5, V6, aVR, Blue arrows: Irregular J-wave 'Fish-hook' appearance in leads V4 and V5

Unfortunately, due to limited resources, labs were not immediately available. The EKG on admission showed some >1 mm ST-segment elevations in lead I, II, V2-V6, and some PR depressions in lead I, II, V4, V5, and V6 (Figure 1). Point of care ultrasound (POCUS) showed no pericardial effusion, no structural abnormalities, and left ventricle (LV) function was intact visually (Video 1). 

**Video 1:** Point of care ultrasound findings Point of care ultrasound showing no evidence of pericardial effusion.

Treatment

**Figure 2:**
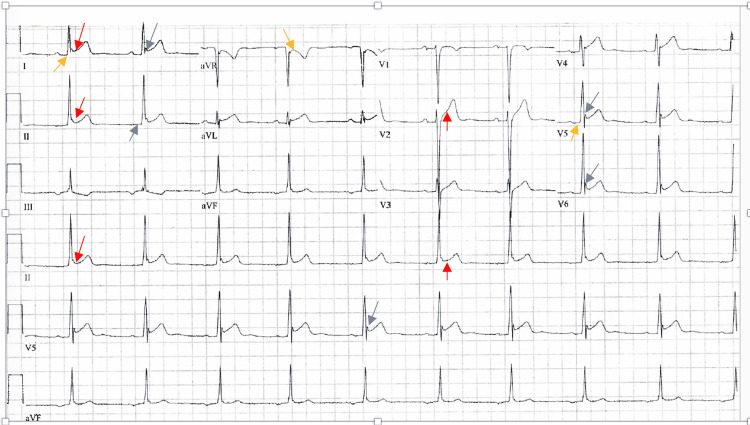
Follow up EKG on day three EKG day three after presentation. Note diffuse ST-segment elevations (red arrows) and PR depressions (orange yellows), 'Fish-hook' patterns (grey arrows), and T-wave inversions in lead III. Marked T-wave amplitudes in V2.

The patient was initially started on a short course of colchicine for a presumed clinical diagnosis of acute pericarditis based on typical symptoms and some EKG findings. However, ER remained a differential. The EKG three days later showed persistent ST-segment changes (Figure 2), and the patient was still symptomatic. A second opinion was found, at a large cost to the patient in both time, and effort as they had to travel far from their underserved community, and it was felt ER alone could explain most of the EKG changes. As a result, colchicine was stopped. 

**Figure 3:**
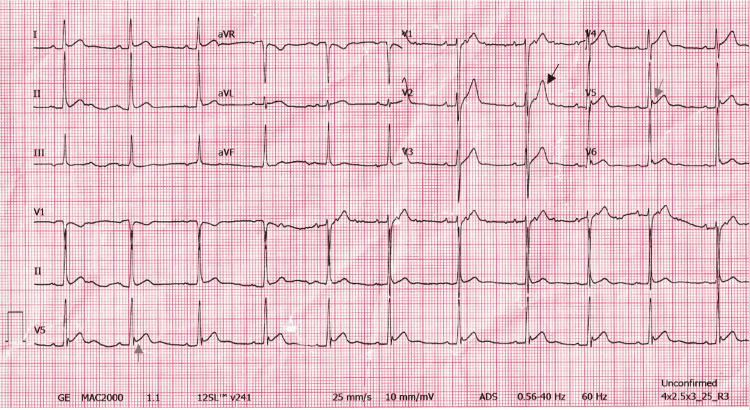
EKG three months post-discharge The EKG three months later, was largely unchanged. Noted were persistent ST-segment elevations and 'Fish-hook' patterns (grey arrows). A marked amplitude of T-wave was still present (black arrow).

The patient was followed up in three months with a repeat EKG and outpatient visit. His symptoms had resolved by then. The EKG at the time showed persistent and consistent ST changes abnormality as well (Figure 3).

## Discussion

Early repolarization is a relatively common finding in young healthy males. It has been thought that the incidence of ER in a population of average aged 25-year-old males is roughly 18%, with most being isolated to the inferior, lateral, or rarely, both. It has also been shown that over 20 years the incidence of ER decreases to roughly one-quarter of what it initially was in the same patients. Early repolarization may hold an increased risk of idiopathic ventricular fibrillation, though it is difficult to gauge how much, and the overall prevalence of this remains very low [16]. These patients often present with a history of syncope or sudden cardiac death (SCD) secondary to ventricular fibrillation arrest. The ER syndrome is often diagnosed by the presence of >1mm J-point elevation in more than two contiguous inferior and/or lateral leads in a survivor of sudden cardiac arrest or patient with unexplained ventricular fibrillation (VF) or polymorphic ventricular tachycardia (VT) [18]. The ER syndrome is very unlikely without a personal/family history of syncope or SCD. It is important to differentiate the ST-segment elevations from ER between Brugada syndrome, pericarditis, and acute myocardial infarction. There are some important characteristics of each to distinguish them. 

Early repolarization vs. ST-elevation MI (STEMI)

It is crucial to distinguish ER from other pathologies with elevated ST segments. Most importantly, acute myocardial infarction also presents with chest pain and most often ST changes on EKG. Several key EKG changes help distinguish between the two. The morphology and distribution of the ST-segment elevation can help clinicians distinguish between the two. As seen in our case, the ST segment rises from a J-point as opposed to a tombstone pattern. It also maintains some level of concavity (as seen above in Figures 1-3). The ST-segment elevation in acute STEMI is usually convex, dome-shaped, and is often greater than 5mm in amplitude. The ST-segment elevations are also confined to anatomical groupings of leads rather than widespread in STEMI patients. Reciprocal ST-segment depression changes are not seen in ER. Other changes like hyperacute T waves, Q waves, and QT prolongation are also not seen in ER.

Early repolarization vs. Brugada syndrome

Brugada syndrome is caused by a genetic mutation that influences the ionic currents in the cardiac muscle of the heart. The EKG changes associated with this include ST-segment elevation in leads V1-V3 with right bundle branch block appearance. There may also be some slowing of the signal as the PR interval may be increased. Typically the ST-segment elevation does have a J-point elevation, which is present in our case, but it is often followed by a negative T wave i.e., (I) a saddlebacked T wave, (II) or a downward sloped elevation to only 1 mm elevation at the terminal end, or (III) EKG changes associated with Brugada can change with exertion, fevers, or medications. Brugada syndrome does not produce ST-segment elevations in other leads as was present in our case. Our case does not exhibit a Brugada-like morphology based on the changes described above. Brugada syndrome is of great clinical significance as it can lead to arrhythmias such as ventricular tachycardia or ventricular fibrillation, and if left untreated could lead to a fatal cardiac arrest. It may often present initially as lightheadedness, presyncope, or syncope [18,19].

Early repolarization vs. acute pericarditis

Early repolarization can easily be confused with acute pericarditis, and thus is an important differential in atypical ECG changes. In a study of 48 patients, it was shown in most cases of acute pericarditis, that ST-segment elevations occur in both the limb leads (I, II, III, aVF, aVR, aVL) and precordial leads (V1-V6) whereas in ER there are typically no ST-segment changes in the limb leads [20]. Furthermore, PR depression and evolutionary changes of ST segments seen in pericarditis are not seen in early repolarization. Most notably, in acute pericarditis, the ratio of ST-segment elevation to T-wave amplitude (ST/T) in V6 is typically greater than or equal to 0.25mV. Additionally, ST/T ratio greater than 0.25 in V4, V5 is also diagnostic of acute pericarditis [1]. There is also a characteristic irregular J-wave also known as the fish-hook pattern that is also associated with ER but not acute pericarditis.

It is possible to have both ER and acute pericarditis. To differentiate between this and just one or the other, having a previous or subsequent EKG is of great use. As is the point of care ultrasound (POCUS). In our case, the EKG from a few months later when the patient was asymptomatic was essentially unchanged from when he was symptomatic. The fact that no pericardial effusion was seen on ultrasound points away from pericarditis, however, effusions may not always be prominent and can be missed on POCUS. The presence of PR depression and ST elevation in leads I, II, and aVF disfavors ER. However, a fish-hook pattern came to be seen in V5, V6 on day one, V4 and V5 on day three, and V5 and V6 a month later. The four stages of EKG changes evolution were not seen, which also makes ER more likely. 

The EKG changes in ER are incidental findings and usually, patients will not present with acute chest pain. It is possible that in our case, the patient did have some costochondritis/musculoskeletal chest pain which would explain his symptoms and the incidental ER pattern noted on EKG. Moreover, the chest tenderness on palpation is not characteristic of acute pericarditis. The patient was actively doing chest press exercises before his symptom onset. 

## Conclusions

Overall, our case demonstrates the importance of knowing the causes of ST-segment elevations and being able to differentiate between them while working in a resource-poor environment. This was not correctly identified at first which led to confusion and difficulty for the patient and his family. Subtle differences in the EKG are important in distinguishing between different etiologies and should be well known and understood. Early repolarization changes on EKG can sometimes be confused with other pathologic causes of ST-segment elevation. Clinicians need to be able to differentiate these changes on EKG. In young patients without significant cardiac risk factors, early repolarization should be high on the differential for ST-segment changes on precordial leads of the EKG. Nonetheless, there have been several cases of SARS-CoV-2 viral illness complicated by acute pericarditis, thus clinicians should rule it out when patients present with chest pain. 
